# Analyses of reported severe adverse events after immunization with SARS-CoV-2 vaccines in the United States: One year on

**DOI:** 10.3389/fpubh.2022.972464

**Published:** 2022-10-13

**Authors:** Halinder S. Mangat, Anwar Musah, Susanne Luedtke, Akheel A. Syed, Boby V. Maramattom, Joel Maruthanal, Arnold Bosman, Patty Kostkova

**Affiliations:** ^1^Department of Neurology, University of Kansas Medical Center, Kansas City, KS, United States; ^2^UCL Centre for Public Health in Emergencies (dPHE), Institute for Risk & Disaster Reduction, Faculty of Mathematics & Physical Sciences, University College London, London, United Kingdom; ^3^Division of Infection Control, COVID-19 Management Group and Vaccine Implementation Team, Public Health Authority, Nuremberg, Germany; ^4^Faculty of Biology Medicine and Health, The University of Manchester, Manchester, United Kingdom; ^5^Department of Neurology, Aster Medcity Hospital, Kochi, Kerala, India; ^6^Department of Neurology, Kansas University Medical Center, Kansas City, KS, United States; ^7^Transmissible BV, Public Health Learning Solutions, Utrecht, Netherlands; ^8^UCL Centre for Public Health in Emergencies (dPHE), Institute for Risk & Disaster Reduction, Faculty of Mathematics & Physical Sciences, University College London, London, United Kingdom

**Keywords:** COVID-19 vaccination adenovector, mRNA, severe adverse events following immunization, BNT-162b2 vaccine, mRNA-1273 vaccine, Ad26.COV2.S

## Abstract

**Objective:**

To analyze rates of reported severe adverse events after immunization (sAEFI) attributed to SARS-CoV-2 vaccines in the United States (US) using safety surveillance data.

**Methods:**

Observational study of sAEFI reported to the vaccine adverse events reporting system (VAERS) between December 13, 2020, to December 13, 2021, and attributed to SARS-CoV-2 vaccination programs across all US states and territories. All sAEFI in conjunction with mRNA (BNT-162b2 or mRNA-1273) or adenovector (Ad26.COV2.S) vaccines were included. The 28-day crude cumulative rates for reported emergency department (ED) visits and sAEFI viz. hospitalizations, life-threatening events and deaths following SARS-CoV-2 vaccination were calculated. Incidence rate ratios (IRRs) of reported sAEFI were compared between mRNA and adenovector vaccines using generalized Poisson regression models.

**Results:**

During the study period, 485 million SARS-CoV-2 vaccines doses were administered nationwide, and 88,626 sAEFI reported in VAERS. The 28-day crude cumulative reporting rates per 100,000 doses were 14.97 (95% confidence interval, 14.86–18.38) for ED visits, 5.32 (5.26–5.39) for hospitalizations, 1.72 (1.68–1.76) for life-threatening events, and 1.08 (1.05–1.11) for deaths. Females had two-fold rates for any reported AEFI compared to males, but lower adjusted IRRs for sAEFI. Cumulative rates per dose for reported sAEFI attributed to adenovector vaccine were 2–3-fold higher, and adjusted IRRs 1.5-fold higher than mRNA vaccines.

**Conclusions:**

Overall cumulative rates for reported sAEFI following SARS-CoV-2 vaccination in the US over 1 year were very low; single-dose adenovector vaccine had 1.5-fold higher adjusted rates for reported sAEFI, which may however equate with multiple-doses mRNA vaccine regimens. These data indicate absence of high risks of sAEFI following SARS-CoV-2 vaccines and support safety equipoise between mRNA and adenovector vaccines. Public health messaging of these data is critical to overcome heuristic biases. Furthermore, these data may support ongoing adenovector vaccine use, especially in low- and middle-income countries due to affordability, logistical and cold chain challenges.

## Introduction

Coronavirus disease 2019 (COVID-19) caused by the severe acute respiratory syndrome novel coronavirus-2 (SARS-CoV-2) was declared a pandemic by the World Health Organization (WHO) in March 2020. Rapid scientific advancements in the management of COVID-19 have been spearheaded by collaborative global efforts, especially in vaccine development utilizing several different platforms, including the mRNA [BNT-162b2 (Pfizer/BioNTech) and mRNA-1273 (Moderna)] and adenovector [ChAdOx1 (AstraZeneca) and AD26.COV2.S (Janssen)] technologies. As of December 13, 2021, 8.5 billion vaccine doses had been administered worldwide, including 485 million in the United States (US) and 1 billion in Europe, though fully vaccinated population proportions remained low at 61% in the US, 59% in Europe, and 46% worldwide (1).

Access remains a key hurdle in low- and middle-income countries (LMICs), whereas vaccine hesitancy is the top reason for low vaccination rates in high-income countries (HICs) (2, 3), even though vaccine safety has been addressed early and methodically in clinical trials (4). However, legitimate concerns of severe adverse events after immunization (sAEFI) (2, 5–8) pose heuristic challenges *via* media narrative, and legitimize vaccine skepticism with incomplete information.

Several severe and life-threatening adverse events, such as thrombosis with thrombocytopenia syndrome (TTS) and cerebral venous sinus thrombosis (CVST) (6, 9), arterial thrombotic events (10), acute demyelinating inflammatory polyneuropathy (Guillain-Barré syndrome, GBS) (7), and myocarditis (11, 12) have been reported as potential SARS-CoV-2 vaccine-related adverse events. Even though causal relationships remain unascertained (13), these occurrences have fueled vaccine skepticism, mistrust and hesitancy (14), affecting specific demographics and minorities (15, 16), who are more likely to suffer severe consequences of COVID-19 (17).

To facilitate the success of vaccine programs and vaccine equity, examining post-clinical trial “real world” surveillance vaccine safety data is necessary to detect possible safety signals which may be too rare to detect even in large clinical trials. This information can empower global health agencies such as the WHO and other policy decision-makers, in the continued planning and implementation of vaccine programs worldwide, as the majorities of global populations remain unvaccinated, while supplies of existing vaccine stocks remain unused in HICs.

In this study, we aimed to identify if composite rates of various vaccine-related illnesses are associated with significant reported rates of hospitalization, life-threatening events and deaths, which are classified as severe adverse events following immunization (sAEFI), using data from the vaccine adverse events reporting system (VAERS) (18).

VAERS is a vaccine safety surveillance registry. Data from it is not meant to provide estimates of true incidence rates of adverse events. Within the spectra of adverse events, there is a wide range of accuracy in reporting (28–72%) (19). However, it has also been demonstrated physicians in hospitals who see severe adverse events are more likely to report them compared to physicians in community practices who see milder adverse events (19). We have therefore selected sAEFI which occur in hospitals, and given the gravity such as hospitalizations, life-threatening events and death, are more likely to be diagnosed and reported. Here we calculate the rates of reported sAEFI from the surveillance system which are not to be conflated with absolute incidence rates. The decisions to define acceptable risks are essentially a task for epidemiologists and taken in relation to risks posed by disease vs. those of prevention and treatments of the disease, not only to individual but also to health systems, and society, keeping in mind that even risks and outcomes of disease themselves may equally be under-reported as has been demonstrated recently with respect to global deaths (20). Furthermore, risk identification and quantification of vaccine-related adverse events such as myocarditis, GBS or CVST, do not present to society an overall risk of vaccines but rather an arbitrary risk which is not easy for individuals to understand in the larger context of a pandemic, and has resulted in significant anxiety. In such circumstances, composite all-cause risks of sAEFI may present a clearer picture, even if it be an estimate and not an absolute risk.

## Materials and methods

### Study design and population

In this observational study, we analyzed data on reported sAEFI viz. hospitalizations, life-threatening events, and deaths following vaccination with each of the three SARS-CoV-2 vaccines (BNT-162b2, mRNA-1273 and Ad26.COV2.S) licensed for emergency use in the US, from the VAERS database between December 13, 2020, and December 13, 2021. All events that occurred up to 28 days after vaccination were included (21).

VAERS is a voluntary adverse event reporting system for all vaccines administered to children or adults, established by the Centers for Disease Control and Prevention (CDC) (22). Healthcare providers are required to report any listed adverse event from the VAERS “Table of Reportable Events”, such as hospitalization, life-threatening event, death, permanent disability, congenital anomaly, or birth defect that occurs following vaccination within a pre-specified time-period, or any similar adverse event listed as a contraindication to further doses of the vaccine. The VAERS registry includes data on demographics, geographical location, date(s) of vaccination, date(s) of adverse event report, symptoms, recovery, disability, and if there is a report that any healthcare was sought; all entries are anonymized, and data is publicly accessible. Unlike absolute risks, sAEFI rates in VAERS are subject to biases. As stated above, whilst reporting rates of all AEFI range widely (28–72%), sAEFI are more accurately recorded by physicians and reported in hospitals, compared to minor AEFIs seen in primary care (19).

### Exposure

The primary exposures of interest were SARS-CoV-2 vaccines, categorized as mRNA (combining BNT-162b2 and mRNA-1273 vaccines) and adenovector (Ad26.COV2.S vaccine). Analyses were also repeated for each mRNA vaccine brand separately (BNT-162b2 and mRNA-1273 vaccines).

### Outcomes

We focused on sAEFI viz. hospitalizations, life-threatening events, and deaths attributed to the SARS-CoV-2 vaccines due to population level implications. In addition, we included emergency department (ED) visits to determine whether increased visits to ED were related to sAEFI, for non-severe events or rather due to WHO categorized immunization-anxiety related reaction related to publications of rare but severe illnesses. These four healthcare outcomes are also less likely to be underreported; adverse events severe enough to warrant a hospital visit are mandated to be reported to VAERS (18).

### Statistical analysis

The VAERS dataset for all AEFI attributed to SARS-CoV-2 vaccines was downloaded, reformatted, and restricted to vaccines administered between December 13, 2020, and December 13, 2021. Duplicate entries and entries with missing vaccination date or manufacturer information were excluded. Data on numbers of 1^st^, 2^nd^, and booster vaccine doses administered were available, but the adverse events are not reported by dose number; therefore, it was not possible to calculate reported event rates per persons or dose sequence, but rather per total doses administered. National vaccine administration demographics and vaccine manufacturer data were obtained from the CDC public access portal (23, 24).

### Determination of cumulative reporting rates

Cumulative reporting rates of each reported outcome were calculated for each vaccine and vaccine type. Rates were calculated as cumulative reported sAEFI per 100,000 administered doses for the 366 days for each vaccine, and 95% confidence intervals (CI) were generated. Additional descriptive analyses included the generation of graphical outputs of temporal trajectories of moving 7-days averages of sAEFI for the three vaccines to visualize timelines of reporting rates.

### Comparing relative rates for sAEFI reporting between vaccines

A generalized Poisson regression model was used to calculate reporting incidence rate ratios (IRRs) for the adenovector (Ad26.COV2.S) vaccine compared to mRNA vaccines (BNT-162b2 and mRNA-1273) for each of the four outcomes (i.e., ED visits, hospitalizations, life-threatening events, and death) with 95% CIs. The model was adjusted for age [grouped as <17, 18–24, 25–39, 40–49, 50–64, >75 years (referent category)] and sex [males and females (referent category)]. Further, interactions between age, sex and types of vaccine were investigated. These results were decomposed using contrasts to reveal the actual effect for all possible pairs. We report the joint effects for male and vaccine-types with the female category treated as the reference group; and the joint-effects for the age groups and vaccine-types with the >75 years category as the reference group. All data management and formatting were carried out in Stata 17 (StataCorp, College Station, TX, United States). All statistical analyses were performed in RStudio (1.4.1717).

We further compared the published rates of serious adverse events that have led to restrictions on the use of different vaccine types, to all-cause rates of reported sAEFI in VAERS. The 3 main events we compared were TTS (6, 9) and GBS (7, 25) following adenovector virus vaccine (Ad26.COV2.S),and myocarditis with mRNA vaccines (BNT-162b2 and mRNA-1273) (8, 11, 12).

## Results

During the study period, 485,359,746 SARS-CoV-2 vaccine doses were administered in the US; 564,108 unique AEFI and 88,626 sAEFI attributed to SARS-CoV-2 vaccinations were reported within 28 days of vaccination (Supplementary Figure 1). Timeline of cumulative doses of each vaccine, vaccine type, and dose sequences, along with cumulative total sAEFI during study period are shown in Supplementary Figure 2.

Median age (interquartile range, IQR) of recipients with any AEFI was 48 (34–63) years; 45 (31–60) years for BNT-162b2, 52 (37–66) years for mRNA1273, and 43 (31–56) years for Ad26.COV2.S groups (Table 1). Females comprised 69.9% of those who reported any AEFI; 69.1% in the BNT-162b2 group, 72.0% in the mRNA1273 group, and 61.8% in the Ad26.COV2.S group.

**Table 1:** Descriptive characteristics.

	**All SARS-CoV-2** ***n* (%)**	**BNT-162b2** ***n* (%)**	**mRNA-1273** ***n* (%)**	**Ad26.COV2.S** ***n* (%)**
Age (years) median (IQR)	48 (34–63)	45 (31–60)	52 (37–66)	43 (31–56)
**Age category (years)**				
≤17	28,876 (5.12)	21,891 (8.52)	5,986 (2.25)	999 (2.44)
18–24	33,283 (5.90)	15,948 (6.20)	12,767 (4.80)	4,568 (11.16)
25–39	129,544 (22.96)	61,570 (23.95)	55,875 (21.00)	12,099 (29.55)
40–49	93,145 (16.51)	44,083 (17.15)	41,317 (15.53)	7,745 (18.91)
50–64	139,130 (24.66)	61,777 (24.03)	65,940 (24.78)	11,413 (27.87)
65–74	76,983 (13.65)	28,753 (11.18)	45,400 (17.06)	2,830 (6.91)
≥75	43,756 (7.76)	16,085 (6.26)	26,579 (9.99)	1,092 (2.67)
Missing	19,391 (3.44)	6,967 (2.71)	12,223 (4.59)	201 (0.49)
**Sex**				
Female	394,620 (69.95)	177,642 (69.10)	191,669 (72.03)	25,309 (61.81)
Male	161,802 (28.68)	76,616 (29.80)	69,781 (26.22)	15,405 (37.62)
Missing	7,686 (1.36)	2,816 (1.10)	4,637 (1.74)	233 (0.57)
**Any adverse events (%)**	564,108	257,074 (45.57)	266,087 (47.17)	40,947 (7.26)
**Doses administered (%)**	485,359,746	282,267,391 (58.15)	185,388,911 (38.19)	17,206,942 (3.54)

Characteristics of patients with any reported AEFI within 28 days of receiving SARS-CoV-2 vaccination between December 13, 2020, and December 13, 2021, inclusive. (Data was obtained from the vaccine adverse events reporting system (VAERS) registry in the United States). (BNT-162b2, Pfizer-Biontech; mRNA-1273, Moderna; Ad26.COV2.S, Janssen).

**Figure 1:**
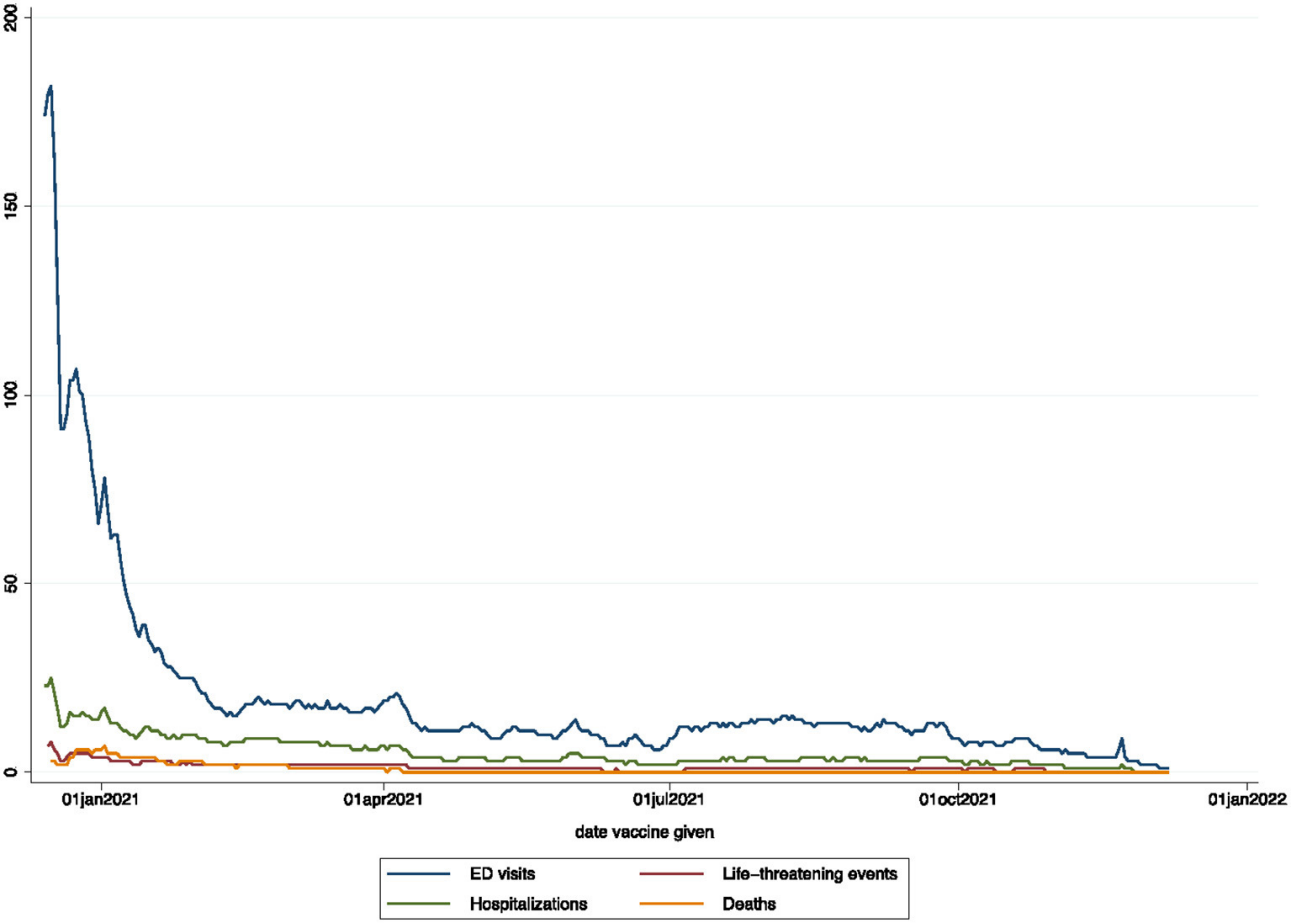
Temporal reporting rates for outcomes (7-day moving average per 100,000 doses).

The overall crude cumulative rate for any reported sAEFI after SARS-CoV-2 vaccination per 100,000 doses was 18.25 (95% CI, 18.13–18.38), 14.97 (14.86–15.08) for ED visits, 5.32 (5.26–5.39) for hospitalizations, 1.72 (1.68–1.76) for life-threatening events, and 1.08 (1.05–1.11) for deaths (Table 2). The averaged reported sAEFI (per 100,000 doses) decreased over the study period (Figure 1). The crude unadjusted rates for sAEFI after adenovector Ad26.COV2.S vaccine were higher than those for mRNA vaccines for each outcome.

**Table 2:** Crude cumulative reporting rates for severe adverse events.

	**Total** **reported** **events** ***n* (%)**	**Crude cumulative** **28-day** **reporting rate** **(95% CI)**
**Any reported adverse event**	**564,108**	
mRNA-1273	266,087 (47.16)	143.52 (142.98–144.07)
BNT-162b2	257,074 (45.57)	91.07 (90.72–91.42)
Ad26.COV2.S	40,947 (7.25)	237.90 (235.66–240.27)
**Any reported severe adverse event**	**88,626 (15.71)**	**18.25 (18.13–18.38)**
mRNA-1273	34,512 (38.94)	18.61 (18.42–18.81)
BNT-162b2	45,990 (51.89)	16.29 (16.14–16.44)
Ad26.COV2.S	8,124 (9.16)	47.21 (46.18–48.24)
**ED visit**	**72,676 (12.88)**	**14.97 (14.86–15.08)**
mRNA-1273	27,455 (37.77)	14.80 (14.63–14.98)
BNT-162b2	38,571 (53.07)	13.66 (13.52–13.80)
Ad26.COV2.S	6,650 (9.15)	38.64 (37.71–39.57)
**Hospitalization**	**25,846 (4.58)**	**5.32 (5.26–5.39)**
mRNA-1273	10,382 (40.16)	5.60 (5.49–5.70)
BNT-162b2	13,132 (50.80)	4.65 (4.57–4.73)
Ad26.COV2.S	2,332 (9.02)	13.55 (13.00–14.10)
**Life-threatening event**	**8,370 (1.48)**	**1.72 (1.68–1.76)**
mRNA-1273	3,268 (39.04)	1.76 (1.70–1.82)
BNT-162b2	4,155 (49.64)	1.47 (1.42–1.51)
Ad26.COV2.S	947 (11.31)	5.50 (5.15–5.85)
**Death**	**5,262 (0.93)**	**1.08 (1.05–1.11)**
mRNA-1273	2,553 (48.51)	1.37 (1.32–1.43)
BNT-162b2	2,272 (43.17)	0.80 (0.77–0.83)
Ad26.COV2.S	437 (8.30)	2.54 (2.30–2.77)

Crude cumulative 28-day reporting rate of severe adverse events occurring within 28 days after SARS-CoV-2 vaccination, that were reported to VAERS between December 13, 2020, and December 13, 2021, inclusive. Reporting rate is expressed per 100,000 doses. (BNT-162b2, Pfizer-Biontech; mRNA-1273, Moderna; Ad26.COV2.S, Janssen). CI, confidence intervals; ED, emergency department. Bold value means total of the subgroups as per row and column labels.

In the multivariable generalized Poisson regression model adjusted for age, sex and vaccine type, IRRs for reported ED, hospitalization and life-threatening events increased with decreasing age, whereas deaths decreased with decreasing age; males had higher IRRs than females for all reported sAEFI (Table 3). Ad26.COV2.S vaccine was associated with higher IRRs for reported ED visits [1.27 (1.24–1.30)], hospitalizations [1.36 (1.30–1.42)], life-threatening events [1.60 (1.49–1.71)], and death [1.51 (1.36–1.66)] compared to mRNA vaccines.

**Table 3:** Incidence rate ratios for reported outcomes.

	**ED visit**	**Hospitalization**	**Life-** **threatening** **events**	**Death**
	**IRR (95% CI)**	**IRR (95% CI)**	**IRR (95% CI)**	**IRR (95% CI)**
**Age group (years)**				
>75 (referent)	1	1	1	1
65–74	0.85 (0.83–0.88)	0.59 (0.57–0.61)	0.81 (0.75–0.88)	0.31 (0.29–0.34)
50–64	1.20 (1.16–1.23)	0.50 (0.49–0.52)	0.82 (0.77–0.88)	0.18 (0.16–0.19)
40–49	1.45 (1.41–1.50)	0.40 (0.38–0.42)	0.77 (0.71–0.83)	0.07 (0.06–0.08)
25–39	1.66 (1.61–1.71)	0.38 (0.37–0.40)	0.61 (0.57–0.66)	0.05 (0.05–0.06)
18–24	2.68 (2.58–2.78)	0.72 (0.68–0.77)	0.91 (0.81–1.02)	0.06 (0.04–0.08)
≤17	2.62 (2.51–2.73)	1.15 (1.08–1.22)	1.02 (0.89–1.17)	0.05 (0.03–0.07)
Missing	0.40 (0.37–0.43)	0.32 (0.30–0.35)	0.25 (0.20–0.30)	0.11 (0.09–0.14)
**Sex**				
Female (referent)	1	1	1	1
Male	1.09 (1.07–1.10)	1.97 (1.92–2.02)	1.90 (1.82–1.99)	2.57 (2.43–2.72)
Missing	0.87 (0.77–0.97)	0.84 (0.68–1.02)	1.08(0.77–1.48)	1.97 (1.41–2.66)
**Vaccine type**				
mRNA (referent)	1	1	1	1
Adenovector	1.27 (1.24–1.3)	1.36 (1.3–1.42)	1.60 (1.49–1.71)	1.51 (1.36–1.66)

Multivariable generalized poisson regression model exploring association between the vaccine type and outcomes, adjusted for age and sex. (December 13, 2020, to December 13, 2021, inclusive). (BNT-162b2, Pfizer-Biontech; mRNA-1273, Moderna; Ad26.COV2.S, Janssen). IRR, Incidence Rate Ratio; CI, confidence intervals; ED, emergency department.

In sex-specific interaction, males had higher reporting IRRs for ED visits, hospitalizations and life-threatening events compared to females with both vaccine-types except for Ad26.COV2.S associated ED visits which were similar (Table 4). When compared to vaccinees older than 75 years, reporting IRRs for ED visits increased with decreasing age in both vaccine groups, while hospitalization and life-threatening events carried a lower reporting IRR in all age groups under 75 years except in the <17 years age group who received mRNA vaccines. Sex adjusted IRRs amongst Ad26.COV2.S group were higher in the 24–49-year group for reported ED visits, but lower for hospitalizations in < 18-year-olds and lower for life-threatening across all ages compared to mRNA vaccines.

**Table 4:** Interactions for outcomes by demographic characteristics.

	**ED visit**		**Hospitalization**		**Life-threatening event**	
	**mRNA**	**Adenovector**	**mRNA**	**Adenovector**	**mRNA**	**Adenovector**
**Age groups** (years)						
>75 (referent)	1	1	1	1	1	1
65–74	0.85^†^	1.01	0.59^†^	0.59^‡^	0.82^‡^	0.69^†^
50–64	1.21^‡^	1.19^*^	0.51^‡^	0.45^‡^	0.85^‡^	0.57^‡^
40–49	1.45^‡^	1.64^‡^	0.40^‡^	0.36^‡^	0.80^‡^	0.50^‡^
25–39	1.64^‡^	2.05^‡^	0.39^‡^	0.34^‡^	0.64^‡^	0.41^‡^
18–24	2.57^‡^	4.04^‡^	0.76^‡^	0.42^‡^	0.99	0.39^‡^
≤ 17	2.62^‡^	0.88	1.16^‡^	0.12^†^	1.06	0.18
**Sex**						
Female (referent)	1	1	1	1	1	1
Male	1.1^‡^	0.98	2.04^‡^	1.43^‡^	1.94^‡^	1.63^‡^

Post-hoc analysis and decomposition of the interaction terms derived from the generalized multivariable Poisson regression model for vaccine-type with sex and age groups. Interaction model for death failed to converge due to very low sample points.

(^‡^p < 0.001;

† 0.001 < p < 0.01;

* 0.01 < p < 0.05). mRNA vaccine type, mRNA-1273 and BNT-162b2 vaccines, Adenovector vaccine type, Ad26.COV2.S. (BNT-162b2, Pfizer-Biontech; mRNA-1273, Moderna; Ad26.COV2.S, Janssen). ED, emergency department.

## Discussion

Our study examines sAEFI reported and attributed to vaccination with SARS-CoV-2 and provides data on the absence of significant risk of such events from a vaccine safety surveillance database and is therefore of great public health importance. The data demonstrate that reports of sAEFI rates are very low compared to the corresponding risks from COVID-19 as well as historic data.

The cumulative rates of reported hospitalizations, life-threatening events, and deaths attributed to SARS-CoV-2 vaccines and occurring within 28 days of vaccination were 5.3, 1.7, and 1.1 per 100,000 doses, respectively (the latter two risks are lower than the risk of dying in 100,000 h of flying). While these are not absolute incidence rates and should not be so conflated, surveillance data from VAERS indicates an overall absence of significant rates of sAEFI which would alert regulators of serious safety concerns. These results are comparable to those published by the European Medicines Agency (EMA) from the EudraVigilance database as of August 29, 2022, with a risk of death from BNT-162b2 reported to be 1.23/100,000 doses, for mRNA-1,272 at 0.71/100,000 doses, and Ad26.COV2.S at 1.71/100,000 doses (26).

Crude reporting rates per 100,000 doses for the adenovector Ad26.COV2.S vaccine seem higher than those for mRNA vaccines; however, it is important to note that Ad26.COV2.S is a single dose regimen for initial immunization, whereas the mRNA vaccine regimen would expose vaccinees to this risk twice, making the overall risks similar. These are further borne out in the interactions in Table 4 wherein the reporting rates while higher for ED visits in younger age groups, do not appear to translate into higher rates of hospitalizations or life-threatening events and may reflect increased anxiety associated with reported adverse events in these groups prompting more ED visits (immunization anxiety related disorder). Meanwhile, the lower relative rate of sAEFI attributed to SARS-CoV-2 vaccination for females compared to males may be due to a higher baseline age-specific mortality rates in males for every adult age stratum (numerator) (27), or because of the lower total number of reported adverse events among males (denominator): the latter of which is supported by evidence of sociocultural barriers that prevent males from seeking medical services (28). However, this requires further investigation.

The overall risks of sAEFI (hospitalization, life-threatening illness, death) for new vaccines are reported to be up to 7% in the literature, with clear early over-reporting (18). In our findings, the reporting rates are much lower than this threshold, and the initial high reporting rates mirror the expected reporting pattern. However, initial high reporting rates of sAEFI may also be linked to the populations selected to be vaccinated earlier: older, nursing home populations who are more vulnerable. Additionally, the outcomes rates may also include unrelated background population event rates; approximately 723 deaths per 100,000 people occur annually in the US, as well as those related to COVID-19 disease in those experiencing these events in the 14 days after vaccination; therefore, some deaths may have occurred unrelated to the vaccine (27). Similarly, all AEFIs related to vaccines may not be reported completely, though sAEFI occur in hospitals and carry greater reporting accuracy (18, 19).

Clearly recognized illnesses that appear to be related to the vaccines are GBS, TTS and myocarditis. The unadjusted incidence rate of GBS attributed to Ad26.COV2.S in the first 21 days following vaccination was 32.4 (95% CI: 14.8–61.5) per 100,000 person-years, compared to 1.3 (95% CI: 0.7–2.4) per 100,000 person-years attributed to mRNA vaccines, and a between vaccine type groups adjusted relative risk of 20.56 (95% CI: 6.94–64.66) (25). However, the calculated rates are based on 11 confirmed cases from 483,503 recipients of Ad26.COV2.S. of which 8 met Brighton diagnostic criteria level 1 or 2. Similarly, no increase in arterial thrombosis was seen following Ad26.COV2.S, but a nearly two-fold increase in venous thromboembolism with an excess of 29 instances per 100,000 vaccinations, and an excess of 2.5 instances of CVST per 100,000 vaccinations; however, there were 29 fewer deaths than expected (background rate and not reduction from COVID mortality) per 100,000 vaccinations (10). Meanwhile, 1,626 cases of myocarditis were reported in relation to 354 million mRNA vaccine doses, 98% of whom had troponin elevation, 96% were hospitalized and 98% discharged home, in addition to having a shorter course of illness compared to viral myocarditis (8). It would appear that while there is a several fold increase in these illnesses following SARS-CoV-2 vaccinations, the background rate is very low and make the absolute increase in incidence not alarming. If at all rates should be compared it should be between disease-related morbidity vs. protection and risk.

With new variants emerging and a gradual rise in R0 value of the virus, it would appear unsafe to assume some population subgroups such as young children may be not vulnerable to COVID-19, and the risk assessment remains dynamic (29). Furthermore, vaccines not only mitigate risks of disease but also “long-COVID” syndrome (30). Therefore, immunization of the population remains central to the control of the SARS-CoV-2 pandemic. Modeling data suggest that every 1% increase between 40–50% vaccination coverage in 270 days (70% vaccine efficacy) can avert 1.5 million cases, 56,240 hospitalizations, 6,660 deaths, gain 77,590 QALYs, save $602.8 million in direct medical costs, and $1.3 billion in productivity losses (31). Expediting to 180 days could save an additional 5.8 million cases, 215,790 hospitalizations, 26,370 deaths, 206,520 QALYs, $3.5 billion in direct medical costs, and $4.3 billion in productivity losses.

Adverse effects to the SARS-CoV-2 vaccines reported to VAERS in the US have been described by diagnoses as minor and severe AEFIs as well as their distribution frequencies within all AEFIs as well as reporting odds ratios (32). While this is very useful information for scientists and public health professionals, the consumers (the public) are likely to benefit from the estimates of reporting sAEFIs, which we endeavor to provide such that the lay public may be able to have an idea of how rare a severe AEFI may be compared to some daily activities, which can be key in overcoming vaccine hesitancy.

Estimates comparing the overall relative risk of vaccine types are important for international decision making and vaccine confidence. Globally, the death toll of COVID-19 is estimated to be 18 million, yet the production of Ad26.COV2.S has ceased and the US Food and Drug Administration (FDA) has restricted the authorization of AD26.COV2.S only to adults for whom other approved vaccines are not accessible or clinically appropriate (33). Vaccine needs remain immense, especially in low- and middle-income countries, where adenovector vaccines are likely to be most utilized due to low-cost and cold-chain logistics. Policy decisions to restrict adenovirus vaccination in countries with a choice of vaccine types may be feasible in HICs but affects vaccine confidence globally.

The reporting rates of sAEFI highlighted in this analysis is in context to the first 2 and possibly 3 doses of vaccines. Given however that the risk of disease with new variants and immune escape is dynamic, it is difficult to estimate a static risk/benefit of these vaccines.

No perfectly safe vaccine exists; disease control efforts consider risks of treatment or prevention of diseases vs. risks of disease to individual health, health systems and society. There is no clearcut threshold as to what an acceptable risk is, but in view of the mortality of 18 million globally, health system collapses, lockdowns, and harm to economies from COVID-19, the threshold of all-cause acceptable risks must be crystallized as much as possible by epidemiologists, health organizations and governments. For this purpose, these data provide crucial risk information to address existing heuristic biases. Furthermore, clear public health messaging of these risks-benefits of vaccines are imperative.

### Limitations

While VAERS is a well-established reporting system with mandatory reporting of sAEFI for healthcare providers, with the largest dataset of vaccine adverse events in the world, it is a voluntary and passive reporting system and may not capture every sAEFI, nor can every sAEFI be determined to be vaccine related. VAERS collects data on all adverse events in the time following vaccination: adverse events such as death may be coincidental, unrelated, and even possibly related to COVID-19 in those who were incompletely vaccinated or contracted disease within 14 days of immunization, rather than vaccine-related, and therefore causality cannot be inferred, and reporting rates not conflated with true incidence rates. Diagnostic confirmation of entries in large surveillance databases is infeasible, hence actual association with vaccination cannot be certain, nor can this population-level data be used to identify individual risks. Reports of adverse events in VAERS are also unverified and may contain information that is incomplete or inaccurate. Under-reporting may also result in under-estimation of the adverse events in this study. However, in such a large dataset, under-reporting is unlikely to vary significantly by vaccine type, therefore while overall reported rates may be different from true incidence rates, the comparison between vaccines is less likely to be susceptible to these biases. Lastly, the data on sAEFI in this manuscript are reported as per dose as in VAERS the AEs are not reported by dose number. However, it has by now been established that the risks for sAEFI may vary by dose number, thus this remains a limitation in this analysis, as the sAEFI estimates are aggregated across different dose numbers and combination types. Nor are other individual characteristics that may predispose one to be administered a particular vaccine type or be susceptible to sAEFI included in the database.

## Conclusions

Overall rates of reported hospitalizations, life-threatening events and deaths occurring within 28 days of vaccination and attributed to SARS-CoV-2 vaccines in the United States are very low. While adenovector Ad26.COV2.S vaccine appears to carry greater rates for these outcomes; when estimated per individual, the required multiple doses of mRNA vaccines would appear to equate the risks. These results provide population level safety data and equipoise, and support continued use of adenovector vaccine especially in resource-constrained health systems due to low cost and cold-chain requirements. These results provide absence of concerning risks of SARS-CoV-2 vaccines at a population level and appear reassuring for continued vaccination rollout to control COVID-19 related disease. Public health messaging and media dissemination of such data is crucial to maintain public enthusiasm, confidence for vaccine uptake and diminish vaccine hesitancy.

## Data availability statement

Publicly available datasets were analyzed in this study. This data can be found here: https://data.cdc.gov/Vaccinations/COVID-19-Vaccination-Demographics-in-the-United-St/km4m-vcsb/data.

## Ethics statement

Ethical review and approval was not required for the study on human participants in accordance with the local legislation and institutional requirements. Written informed consent for participation was not required for this study in accordance with the national legislation and the institutional requirements.

## Author contributions

Conceptualization: HM, AS, SL, and PK. Data acquisition: HM. Data analysis: AM, HM, and PK. Data interpretation: HM, AM, AS, AB, and PK. Writing manuscript: HM, AM, AS, BM, SL, PK, and AB. Reviewing final manuscript: all authors. All authors contributed to the article and approved the submitted version.

## Conflict of interest

Author AB was employed by the company Transmissible BV. The remaining authors declare that the research was conducted in the absence of any commercial or financial relationships that could be construed as a potential conflict of interest.

## Publisher's note

All claims expressed in this article are solely those of the authors and do not necessarily represent those of their affiliated organizations, or those of the publisher, the editors and the reviewers. Any product that may be evaluated in this article, or claim that may be made by its manufacturer, is not guaranteed or endorsed by the publisher.
