# Supplementary figures and images for: Analyses of reported severe adverse events after immunization with SARS-CoV-2 vaccines in the United States: One year on
Source: Front Public Health. 2022 Oct 13;10:972464. doi: 10.3389/fpubh.2022.972464 (PMC9610110; doi:10.3389/fpubh.2022.972464)

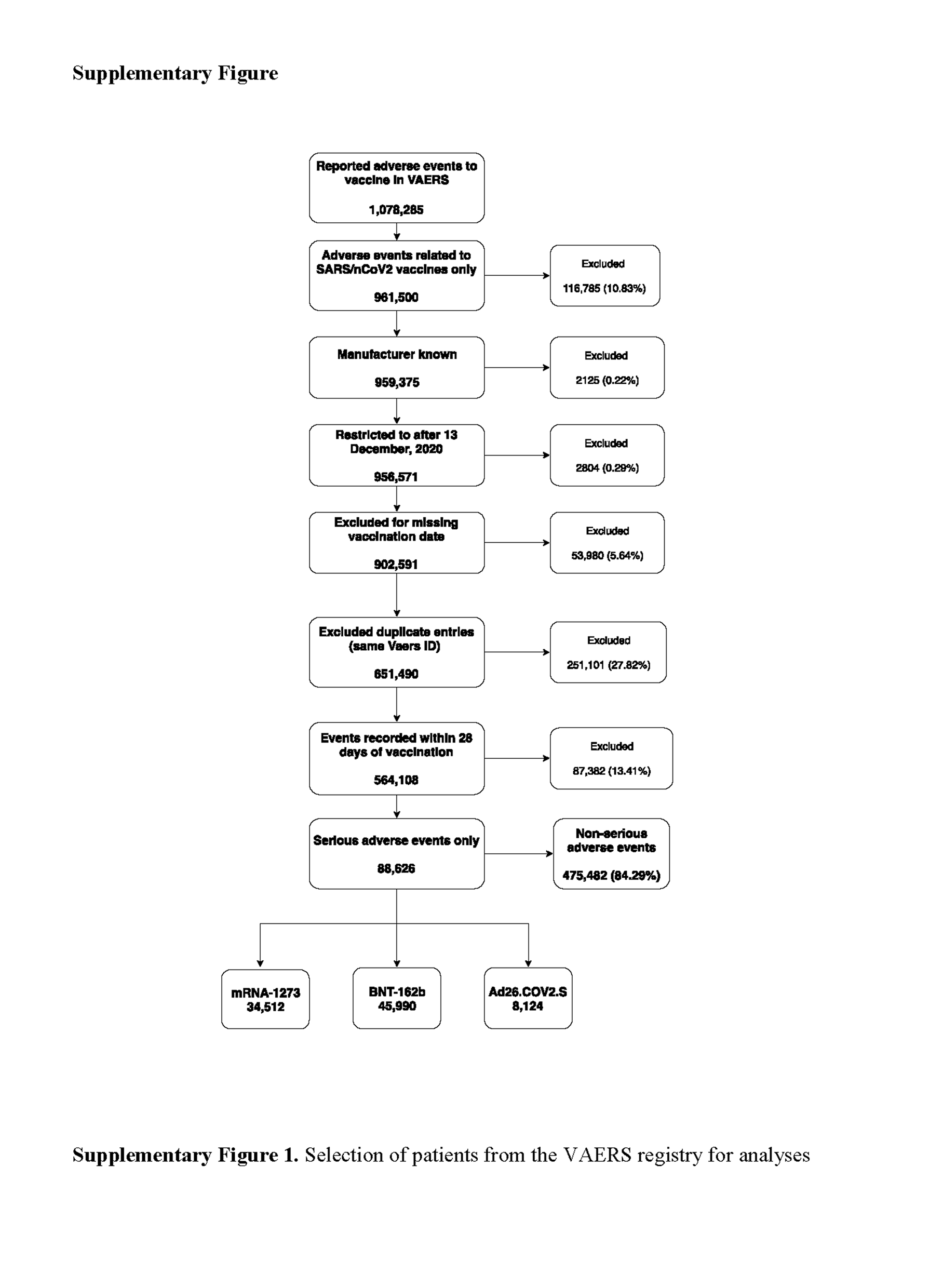

Supplement: Supplementary file 1 [file Image_1.tiff]

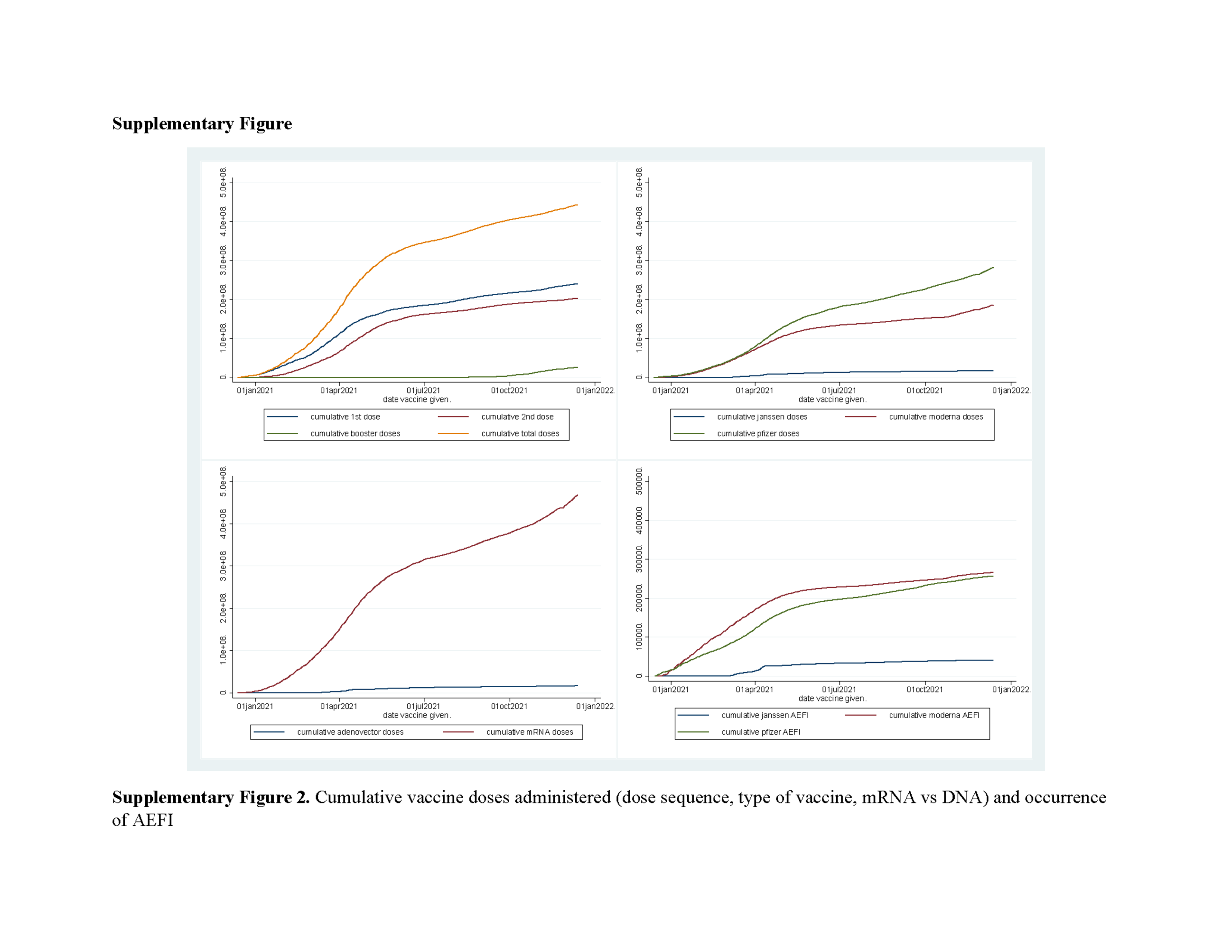

Supplement: Supplementary file 2 [file Image_2.tiff]
